# In Vitro Evaluation of *Thymus serpyllum* Essential Oil Against *Paracoccus marcusii* IBR3: A Potential Natural Protection Strategy for Historic Mural Paintings

**DOI:** 10.1111/1758-2229.70368

**Published:** 2026-05-29

**Authors:** Michele Narduzzi, Claudia Pelosi, Vittorio Vinciguerra, Chiara Antonelli, Anna Maria Vettraino

**Affiliations:** ^1^ Department for Innovation in Biological, Agro‐Food and Forest Systems (DIBAF) University of Tuscia Viterbo Italy; ^2^ Department of Economics, Engineering, Society and Business Organization (DEIM University of Tuscia Viterbo Italy

**Keywords:** bacteria, biodeterioration, cultural heritage, mural paintings, *Paracoccus*, thyme essential oil

## Abstract

Historic mural paintings are particularly vulnerable to biodeterioration caused by microbial colonization, which can lead to the degradation of the organic and inorganic components of wall surfaces. This study presents, to the best of our knowledge, the first identification of the *Paracoccus marcusii* IBR3 strain on a 19th‐century mural painting from a private Italian villa. In vitro analyses revealed that strain IBR3 exhibits significant enzymatic activities, such as casein hydrolysis and calcium carbonate dissolution. These activities implicate the strain in the degradation of proteinaceous binders and mineral substrates, which are typical of mural artwork. As part of an environmentally sustainable conservation strategy, the antimicrobial activity of *Thymus serpyllum* essential oil was evaluated against strain IBR3 using in vitro tests, membrane integrity assays, and ATR‐FTIR spectroscopy. The essential oil, chemically characterized by GC–MS, demonstrated promising biocidal efficacy. These findings suggest that *T. serpyllum* essential oil could serve as a non‐toxic alternative for preserving and maintaining culturally significant wall paintings.

## Graphical abstract

Historic mural paintings are highly vulnerable to microbial biodeterioration. This study reports the first identification of *Paracoccus marcusii* strain IBR3 on a 19th‐century Italian mural. In vitro tests revealed enzymatic activities, including casein hydrolysis and calcium carbonate dissolution, linking the strain to protein binder and mineral degradation. To counteract this, the antimicrobial efficacy of *Thymus serpyllum* essential oil was assessed. Chemically characterized by GC–MS, the oil showed significant biocidal activity, suggesting a sustainable, non‐toxic alternative for mural conservation.

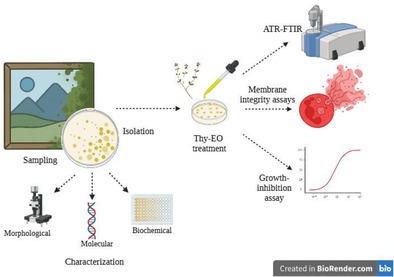

## 1 Introduction

Noble residences, particularly those constructed between the 18th and 19th centuries, symbolize the social prestige of their owners and represent significant cultural and artistic legacies (Godfraind and Manning 2018; Savolainen 2023; Thompson 2023). A key distinctive feature of historical villas is the presence of wall paintings adorning ceilings, rooms, and staircases (Marras et al. 2010; Jiménez‐Desmond et al. 2024). These artworks often illustrate mythological scenes, ornamental motifs, daily life, landscapes, or architectural illusions painted in the trompe‐l'œil style. Preserving them is crucial for heritage conservation, as they are integrated into the architecture itself and often provide insights into the lives, tastes, values, and cultural environment of their time (Pei et al. 2023; Suphaphimol et al. 2022). Fluctuations in humidity and temperature, light exposure, air pollution, and colonization by microorganisms and insects can lead to irreversible damage of these artworks, including fading, cracking, peeling, and discoloration (Gonzalez et al. 1999; Saiz‐Jimenez 1999; Pavić et al. 2015; Gorbushina et al. 2004; He et al. 2021). The complexity of the process of biodeterioration requires a comprehensive understanding of microbial community dynamics, functional diversity, and environmental context. In the micro‐ecosystem established on the mural surface, each member plays a specific role. For instance, cyanobacteria provide organic compounds for heterotrophic bacteria and fungi (Hoffmann 2003; Kosznik‐Kwaśnicka et al. 2022). Meanwhile, fungi release nutrients that can further support microbial growth. Additionally, the hyphal structure of fungi can physically alter the surface by increasing porosity and creating micro‐niches where other microbes can settle and thrive. Understanding the ecological processes that drive microbial colonization and deterioration of mural paintings is essential for selecting targeted and safe antimicrobial strategies.

The growing recognition of microbial impact on mural paintings highlights the urgent need for sustainable and eco‐friendly preservation strategies. Plant extracts gained increasing attention for their ability to inhibit the growth of several fungal and bacterial strains without compromising plants health and cultural heritage materials (Nazzaro et al. 2017; Geweely et al. 2019; Senbua and Wichitwechkarn 2019; Palla et al. 2020; Albasil et al. 2021; Liao et al. 2021; Vettraino et al. 2022; Zikeli et al. 2023; Isola et al. 2025; Mohamed et al. 2025). Essential oils are complex mixtures of bioactive compounds, such as terpenes, phenolics, and aldehydes, that act as antimicrobials by disrupting microbial cell membranes, interfering with enzymatic activity, and inhibiting biofilm formation. Extracts of *Rosmarinus officinalis* exhibited antimicrobial activity against several fungal strains, such as *Aspergillus clavatus*, and bacterial strains, including *Arthrobacter globiformis* and *Bacillus cereus* , at low concentrations (Corbu et al. 2021, 2022). *Thymus serpyllum* , commonly known as wild thyme, is also well known for its bactericidal and fungicidal activity, primarily due to high concentrations of thymol and carvacrol (Pruteanu et al. 2018; Vettraino et al. 2023). Thyme is a low‐growing, aromatic perennial plant belonging to the Lamiaceae family. Thyme essential oil has a long history of use dating back to Egyptian, Roman, and Greek civilizations, primarily for medicinal purposes, and is now also gaining relevance in food conservation practices.

The present study aims to: (1) characterize the bacterial strain IBR3, isolated from a deteriorated mural painting; (2) evaluate the capacity of *T. serpyllum* essential oil to inhibit its growth in in vitro test; and (3) investigate the essential oil's mode of action on bacterial growth.

## 2 Materials and Methods

The research design involved: (a) isolating and characterizing a bacterial strain from a deteriorated mural painting through molecular and biochemical analyses; (b) chemically profiling *T. serpyllum* essential oil via GC–MS and evaluating its antimicrobial effects through growth inhibition assays; and (c) investigating the mode of action of thyme essential oil using membrane integrity tests, permeability assessments, and ATR‐FTIR spectroscopy.

### 2.1 Bacterial Strains Characterization

Samples were collected in August 2024 from a mural painting located in the main room of a private villa, dated late 1800s, in Central Italy. The painting depicted a rural landscape. Sampling was conducted only after authorization was obtained from the property owner. Samples were collected in three small square plots (10 cm^2^) with evident signs of deterioration, due to pink patina, by means of sterile swabs, and avoiding intact, visually significant paint layers. Sampling points were selected in consultation with a qualified conservator. The swabs were combined in the same tube to make a composite sample. Bacteria were isolated using serial dilution techniques, and the taxa were cultured on Tryptic Soy Agar (TSA) medium, at 28°C for 24–48 h.

Single orange colonies were selected from plates at a 10^−3^ dilution and sub‐cultured for 24–48 h. Strains were grouped according to their morphologies. Strain IBR3 was chosen as the representative isolate because it was the most abundant among the recovered isolates and was subsequently used in the following analyses. The bacteria were stained by the Gram method and observed by Nikon Eclipse Ei trinocular optical microscope (NIKON, Osaka, Japan) (Coico 2006). The motility test was carried out using the protocol described by Reller and Mirrett (1975). Bacterial growth on different carbon sources was assessed following the method of Maheswaran and Forchhammer (2003). A minimal M9 medium was used with one of the following different carbon sources, each at 0.4%: d‐glucose, d‐fructose, d‐sucrose, d‐mannose, d‐mannitol, l‐arabinose and d‐sorbitol. The proteolytic activity was evaluated using Milk Nutrient Agar (Milk‐NA) and R2A Agar media, supplemented with 0.4% gelatin, as described by Kraková et al. (2012). Starch hydrolysis was tested using the method of Dhawale et al. (1982). Calcium carbonate (CaCO_3_) dissolution was tested on agar plates following the protocol by Cacchio et al. (2004), while organic acid production was evaluated in Czapek‐Dox liquid medium, as described by Jurado et al. (2021). Indole production from tryptophan was evaluated using the protocol of Antonelli et al. (2025), while urease activity was tested in Urea Agar according to Hussain Qadri et al. (1984).

### 2.2 Molecular Identification

A pure IBR3 fresh colony was collected from a TSA plate using a sterile syringe needle and transferred into the already prepared PCR reaction mixture (Woodman et al. 2016). The PCR amplification of the 16s rRNA genes was done using 27F (5′‐AGAGTTTGATCCTGGCTCAC‐3′) and 1492R (5′‐CGGTTACCTTGTTACGACTT‐3′) primers, according to Sharma et al. (2008). After the quality check, PCR products were sequenced by Eurofins Scientific (Milan, Italy) and blasted against the NCBI database. Phylogenetic trees were reconstructed using the neighbour‐joining algorithm with the Kimura 2‐parameter, implemented in MEGA 11 software (Kimura 1980; Tamura et al. 2021). Bootstrap analysis was based on 1000 replications. Sequences of other *Paracoccus* species used in the phylogenetic analysis were retrieved from GenBank (Table 1).

**TABLE 1:** NCBI GenBank ID of *Paracoccus* species used in the phylogenetic analysis.

*Paracoccus* strain	NCBI ID
*P. carotinifaciens* E‐396	NR_024658
*P. haeundaensis* BC74171	NR_025714
*P. marcusii* zzx35	KJ009431
*P. carotinifaciens* X11	JX122614
*P. seriniphylus* MBT*‐*A4	NR_028968
*P. onubensis* 1011MAR3C25	NR_179420
*P. homiensis* DD*‐*R11	NR_043733
*P. aestuarii* B7	NR_044342
*P. marinus* KKL*‐*A5	NR_041234
*P. thiocyanatus* THI 011	NR_025858
*P. yeei* G1212	NR_029038
*P. cavernae* 0511ARD5E5	NR_149299
*P. kocurii* JCM 8400	LC379078
*P. alkenifer* A 901/1	NR_026424
*P. marcusii* IBR3	PZ013937

### 2.3 Essential Oil of *Thymus serpyllum*

The essential oil of *T. serpyllum* (Thy‐EO) was provided by Inalme SRL (Catania, Italy; Batch number 0E0399). Thy‐EO was submitted for GC–MS analysis, using the Konik instrumentation equipped with a 5000C gas chromatograph and the mass spectrometer detector MS Q2 (Sant Cugat del Vallés, Barcelona, Spain). The gas chromatograph was equipped with a Macherey Nagel (Düren, Germany) fused‐silica column (60 m × 0.25 mm i.d. OPTIMA WAX, film thickness 0.25 μm) and operated under the following conditions: injector temperature at 220°C; transfer line at 240°C; oven temperature programmed from 60°C to 246°C, at 3°C/min; carrier gas—He at 1.0 mL/min, and electron impact (EI) mode at 70 eV with a scan range from 45 to 450 amu (2 scan/min). Identification of the components was performed by comparing their mass spectra with those stored in the NIST 20 computer library and by evaluating the linear retention indices (LRIs) calculated against those available in the literature (Wallace 1999).

### 2.4 Growth Inhibition Assays

The microbial growth of the strain IBR3 was evaluated using the 96‐well plate microdilution assay. A stock solution of *T. serpyllum* essential oil (Thy‐EO) was prepared by dissolving 40 mg of the oil in 0.5% (v/v) Tween 80 and adjusting the final volume to 2 mL with sterile Tryptic Soy Broth (TSB), yielding a final concentration of 20 mg/mL. The stock solution was used at final concentrations ranging from 0.1 to 1 mg/mL.

Serial twofold dilutions of the Thy‐EO stock were made with TSB to yield final concentrations ranging from 0.1 to 1 mg/mL. Positive (A: TSB plus bacterial cells, 20 μL; 9 × 10^7^ CFU/mL; B: TSB, 0.5% Tween 80 and bacterial cells, 20 μL; 9 × 10^7^ CFU/mL) and negative controls (C: TSB) were included at 28°C, with orbital shaking at 150 rpm. After 12 h, the results were measured using a SpectraMax ABS PLUS UV–VIS spectrophotometer (OD_600_ nm).

The MIC value was defined as the lowest concentration of Thy‐EO that inhibited the growth of the microorganism. To evaluate the MBC, a volume of 10 μL of each well was spread onto TSA and incubated aerobically at 28°C for 24 h. The MBC was defined as the concentration with no visible growth on the plate. Positive controls were not treated. The effective dose (EC50) value, which is the concentration of a drug that gives half‐maximal response, was calculated by probit analysis using the Graphpad Instat software (San Diego, CA, USA) (Finney 1971).

### 2.5 Membrane Integrity of IBR3

The integrity of the IBR3 cell membrane was investigated by measuring the absorbance of cellular constituents at 260 nm and 280 nm as described by Cao et al. (2019).

IBR3 was treated with different Thy‐EO concentrations, equal to MIC, 2 × MIC, and 4 × MIC values, and incubated at 28°C for 2 h, shaking at 150 rpm. Triton X‐100 1% and TSB were used as positive and negative controls, respectively. At 0 time and after 2 h, the samples were centrifuged at 3000*g* for 10 min, and the OD of supernatants was measured at 260 and 280 nm using a SpectraMax ABS PLUS UV–Vis spectrophotometer.

### 2.6 Crystal Violet Assay for IBR3 Membrane Permeability

The variation in membrane permeability of the IBR3 strain was evaluated using the crystal violet uptake assay, as described by Vaara and Vaara (1981). Bacterial cells grown in TSB to a concentration of 10^8^ CFU/mL were harvested by centrifugation at 4500 × *g* for 5 min at 4°C. The resulting pellet was washed twice with Phosphate Buffered Saline (PBS) (pH 7.4), resuspended in PBS containing Thy‐EO at 0.4 mg/mL, and incubated at 28°C for 30 min. Control samples were prepared under identical conditions but without Thy‐EO treatment. After the incubation time, bacterial cells were centrifuged at 9300 × *g* for 5 min and resuspended in PBS added with 10 μg/mL of crystal violet. The suspension was incubated at 28°C for 10 min with gentle shaking, then centrifuged at 13,400 × *g* for 15 min. The absorbance of the supernatant was measured at 590 nm using a SpectraMax ABS PLUS UV–Vis spectrophotometer. The OD of the original crystal violet solution (without bacterial exposure) was taken as 100%.

Crystal violet uptake (%) was determined using the formula: (A_sample/A_control) × 100, where A_sample is the absorbance of the sample and A_control is the absorbance of the original crystal violet solution (Devi et al. 2010; Li et al. 2013).

### 2.7 ATR‐FTIR Spectroscopy

Thy‐EO at different concentrations (0.3, 0.4, 0.8, 1.2 mg/mL) was added to a cell suspension of pure IBR3 culture in exponential phase (OD = 0.4). The treatment was performed for 3 h at 28°C (Devi et al. 2010). Cells were then washed three times with 0.9% NaCl solution and centrifuged at 10,000 × *g* for 2 min and dried at environmental temperature (25°C) (Yang et al. 2023). Untreated IBR3 pure cultures were used as controls. All IR spectra (4000–600 cm^−1^) were obtained by the attenuated total reflection (ATR) technique, using an Agilent FTIR Cary 630 model spectrometer equipped with a diamond ATR sampling accessory. A total of 128 scans with 4 cm^−1^ resolution were taken for each sample. The instrument was equipped with a DTGS (deuterated triglycine sulfate) detector. Reproducibility of the absorption spectra was ensured by performing multiple independent measurements.

### 2.8 Statistical Analysis

All experiments were repeated three times using independent biological replicates, and each biological replicate was analysed in triplicate. Data were presented as means ± standard errors. Normality was assessed using the Shapiro–Wilk test. Since the data met the assumption of normality, one‐way ANOVA followed by Tukey's multiple comparison of means test was performed (*p* ≤ 0.05), using the Graphpad Instat software (San Diego, CA, USA). Principal component analysis (PCA) was performed using the prcomp function of R software version 4.3.1., and score plots (PC1 vs. PC2) were used to visualize sample separation using data in the region 4000–650 cm^−1^.

## 3 Results

### 3.1 Identification and Characterization of the IBR3 Bacterial Strain

IBR3 colonies, cultured on a TSA plate at 28°C for 2days, appeared flat, bright orange, and round‐shaped. Cells were sometimes arranged in clusters and pairs, Gram stain‐negative, with no flagella, and non‐motile. *Paracoccus* strain IBR3 grew on several carbon sources (sucrose, fructose, d‐sorbitol, d‐glucose, l‐arabinose, d‐mannose, d‐mannitol), producing organic acids from d‐glucose. The bacteria hydrolysed gelatin but no starch, while tests for indole production and urease activity were negative (Table 2). *Paracoccus* strain IBR3 also showed proteolytic activity towards casein and calcium carbonate dissolving activity.

**TABLE 2:** Comparison of the properties of the strain IBR3 and closely related strains: *P. marcusii* , *P. haeundaensis* and *P. carotinifaciens* .

Characteristic	*Paracoccus* IBR3	*P. marcusii* MH1T	*P. haeundaensis* BC74171T	*P. carotinifaciens* E‐396
Morphology	Cocci to short rods, 1.0 μm in size, growing in single, pairs or short chains.	Cocci to short rods, 1.0–1.3 μm in size, growing in single, pairs or short chains.	Rod‐shaped, 0.3–0.7 μm in diameter and 0.8–2.5 μm in length.	Rod‐shaped, 0.3–0.75 μm in diameter and 1.0–5.0 μm in length.
Motility	−	−	+	+
Orange to red pigment	+	+	+	+
Growth at 40°C	−	−	−	−
*Growth on:*				
Sucrose	+	+	−	+
Fructose	+	+	−	+
d‐Sorbitol	+	+	−	+
d‐Glucose	+	+	−	+
l‐Arabinose	+	+	+	−
d‐Mannose	+	+	−	+
d‐Mannitol	+	+	−	+
*Production of:*				
Urease	−	−	−	−
Indole from tryptophan	−	−	−	−
Organic acids from d‐glucose	+	+	NR	+
*Hidrolysis of:*				
Starch	−	−	+	−
Gelatin	W	W	NR	−

*Note:* Data for *P. marcusii* were obtained from Harker et al. (1998) and Wang et al. (2018), data for *P. haeundaensis* BC74171ᵀ were from Lee et al. (2004) and data for *P. carotinifaciens* E‐396 were from Tsubokura et al. (1999).

Abbreviations: +, Positive; −, negative; W, weakly positive; NR, not reported.

**FIGURE 1:**
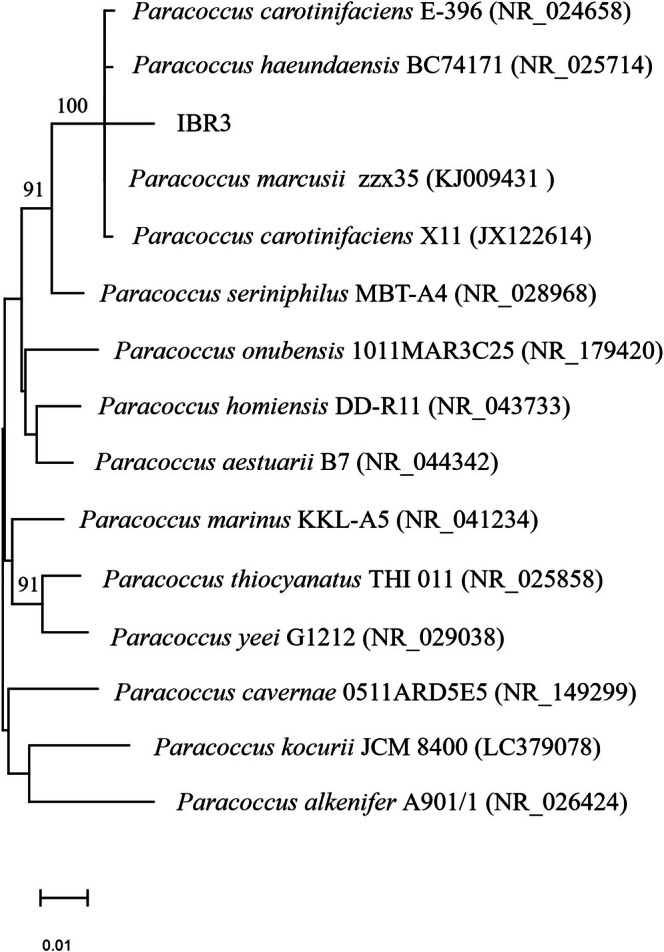
Neighbour‐joining tree based on the 16S rRNA gene sequences, showing the phylogenetic position of strain IBR3 among closely related members of the genus *Paracoccus*. Bootstrap values (based on 1000 replications) greater than 50% are shown at branch points.

Based on morphological, biochemical, and molecular analysis, *Paracoccus* strain IBR3 was identified as *Paracoccus marcusii* IBR3. The sequence of the strain IBR3 was deposited in GenBank with Accession number PZ013937. ITS analysis generated an amplicon with size 1.139 bp, confirming 98.82% sequence identity with *P. marcusii* zzx35 (KJ009431), 99.65% with *P. carotinifaciens* X11 (JX122614), 99.47% with *P. carotinifaciens* E‐396 (NR_024658), and 99.65% with *P. haeundaensis* BC74171 (NR_025714). Phylogenetic analysis, based on 1141 unambiguous bases, revealed that strain IBR3 was a member of the *Paracoccus* genus. It clustered together with *P. marcusii* , *P. haeundaensis* , and *P. carotinifaciens* (Figure 1).

### 3.2 Chemical Composition of *Thymus serpyllum* Essential Oil

Based on the GC–MS analysis, 10 compounds were identified in Thy‐EO. As shown in Table 3, carvacrol (51.88%) and p‐cymene (40.28%) were the dominant components, while thymol accounted for 2.45%. Most compounds were present at concentrations below 1.5%.

**TABLE 3:** Chemical composition (percentage) of *Thymus serpyllum* EO based on the GC–MS analysis.

Components	Relative concentration (%)	LRI^a^	LRI^b^	Retention time (min)
α‐Pinene	0.82	1022	1025	15.082
Fenchene	0.34	1058	1061	15.889
Camphene	2.06	1071	1068	16.018
trans‐p‐Menthane	0.17	1101	n.a.	17.366
β‐Pinene	0.71	1105	1110	17.443
cis‐p‐Menthane	0.22	1118	1122	18.104
p‐Cymene	40.28	1268	1279	19.657
p‐Cymen‐8‐ol	0.09	1805	1818	28.087
Thymol	2.45	2149	2157	32.980
Carvacrol	51.88	2178	2183	33.478

Abbreviation: n.a., not available.

^a^ Linear retention indices measured on polar column.

^b^ Linear Retention Indices from literature (Wallace).

### 3.3 In Vitro Antibacterial Activity of *Thymus serpyllum* Essential Oil

**FIGURE 2:**
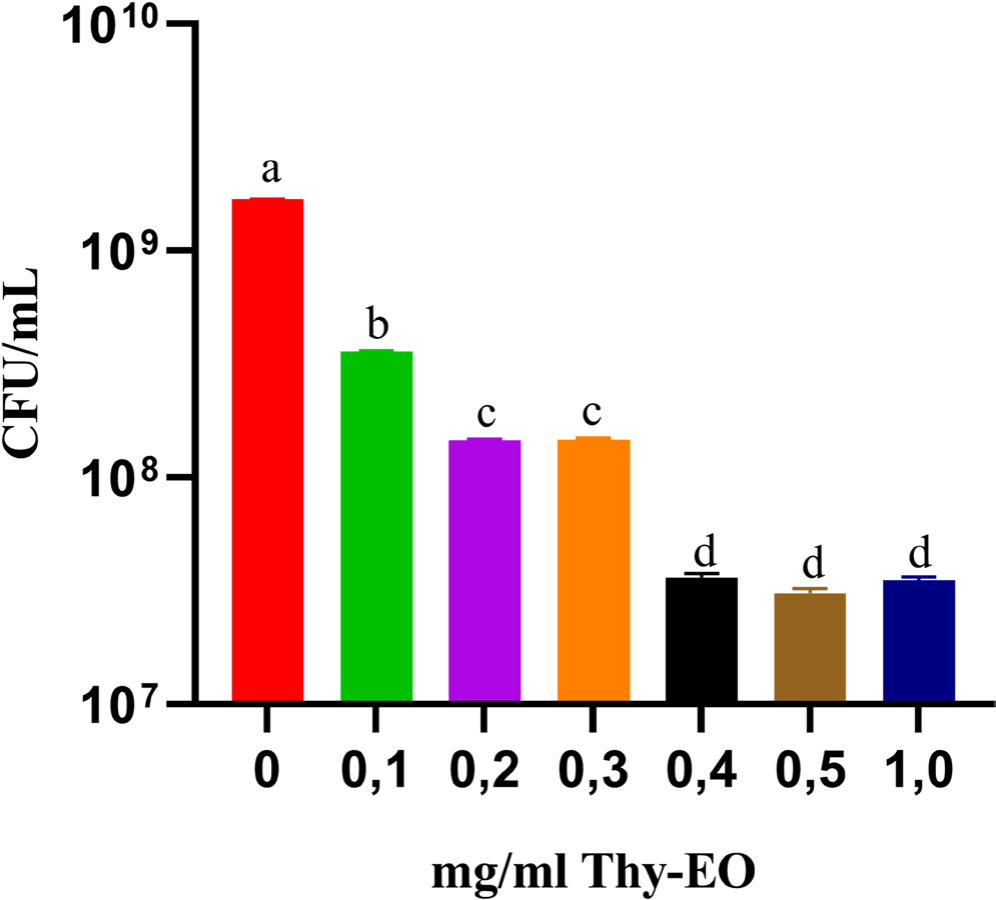
*Paracoccus* strain IBR3 growth at different Thy*‐*EO concentrations, incubation at 28°C for 12 h. Lower case letters represent statistically significant differences among treatments (ANOVA; *p* < 0.05).

The effects of different concentrations (0, 0.1, 0.2, 0.3, 0.4, 0.5, 1.0 mg/mL) of Thy‐EO against IBR3 are shown in Figure 2. Data from the control and tween groups were pooled, as no significant differences were observed between them (Student's *t*‐test; *p* < 0.05). All Thy‐EO doses inhibited bacterial growth. MIC and MBC values were 0.4 mg/mL and 1 mg/mL, respectively. The EC50 value was 0.37 mg/mL.

### 3.4 Cell Membrane Integrity

**FIGURE 3:**
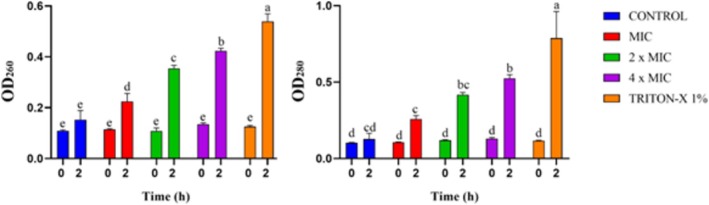
Release of IBR3 UV‐absorbing materials at 260 nm (left) and 280 nm (right) after growth treated with Thy‐EO at MIC, 2× MIC, 4× MIC, and Triton X‐100 (1%), at 0 and 2 h. Lower case letters represent statistically significant differences among treatments (ANOVA; *p* < 0.05).

Treatments with Thy‐EO induced the disruption of the bacterial membrane's integrity. Exposure of bacteria to increasing concentrations of Thy‐EO (MIC, 2 × MIC, and 4 × MIC) resulted in a dose‐dependent increase in the release of both double‐stranded DNA (dsDNA) and intracellular proteins (Figure 3).

### 3.5 Membrane Permeability

The uptake of crystal violet by IBR3 was 21.98% ± 1.23% in the absence of Thy‐EO, while it increased significantly to 26.48% ± 0.25% at 0.4 mg/mL (MIC) of Thy‐EO treatments.

### 3.6 ATR‐FTIR Spectroscopy

**FIGURE 4:**
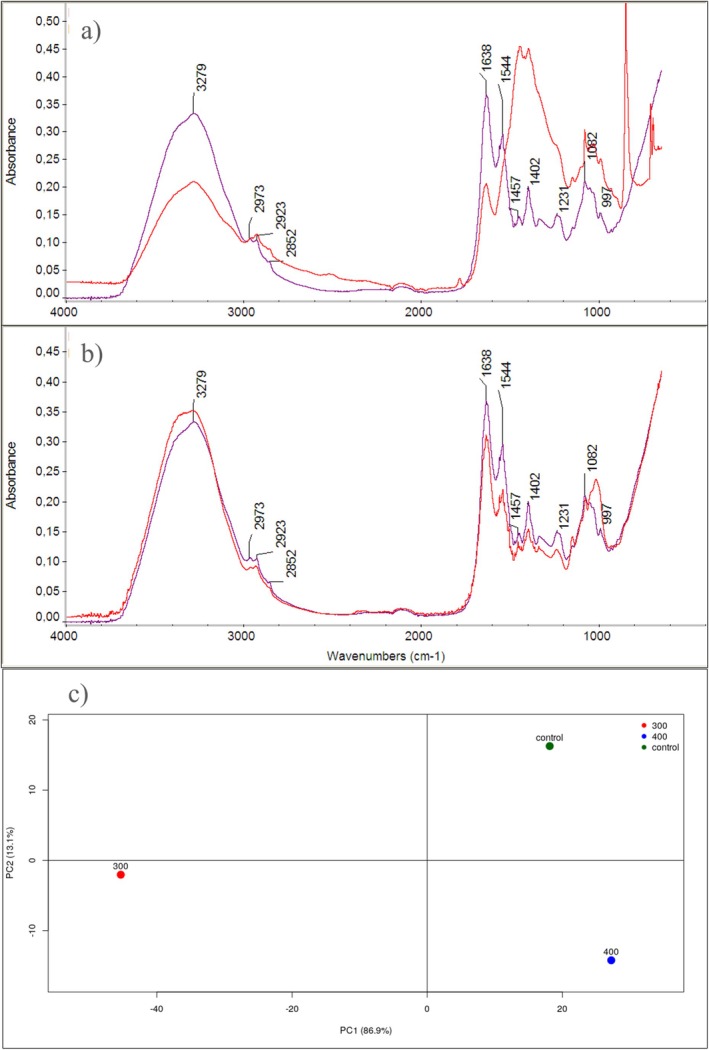
FTIR spectra of IBR3 strain treated with 0.3 mg/mL (a), 0.4 mg/mL (b) of Thy‐EO (red lines), compared to untreated controls (purple lines). (c) PCA plot with loadings (PC1; PC2) of FTIR data in the region 4000–650 cm^−1^.

ATR‐FTIR spectroscopy revealed distinct spectral differences between untreated controls and bacterial cells treated with Thy‐EO. PCA of the FTIR spectra showed a clear separation among the three conditions, with PC1 explaining ~86.9% of the variance and mainly distinguishing the 300 sample from both 400 and control, while PC2 (~13.1%) further separated control from 400 (Figure 4b). The FTIR spectra (Figure 4a) revealed significant differences in the OH stretching region (~3279 cm^−1^) and the CH stretching vibrations at 2973, 2923, and 2852 cm^−1^, and amide I and II bands (1638 and 1544 cm^−1^) between control and treated IBR3 colonies, suggesting alterations in membrane proteins and lipids upon treatment (Aboualizadeh et al. 2017; Kassem et al. 2023). Additionally, changes in peaks at ~1402, 1231, 1082, and 897 cm^−1^ indicate modifications in polysaccharide and phospholipid structures. To ensure correct comparison of the infrared spectra, they have been adjusted to a common scale, using the appropriate tool of Omnic software.

## 4 Discussion

This study expands the known biodiversity associated with mural deterioration, focusing specifically on the culturable bacterial fraction recovered from a deteriorated area. Under the applied culture conditions, *P. marcusii* strain IBR3 was among the most frequently recovered isolates; however, this observation is culture‐dependent and does not necessarily reflect dominance in situ. Nevertheless, to our knowledge, the presence of *P. marcusii* on a deteriorated mural painting in a private villa represents a novel finding in the context of cultural heritage microbiology. This report confirms the role of carotenoid‐producing bacteria in the degradation of mural paintings and, therefore, their contribution to the aesthetic damage in addition to that of the constituent materials (Harker et al. 1998; Gomoiu et al. 2017; Cojoc et al. 2019).

The genus *Paracoccus* encompasses more than 83 different species, which are Gram‐negative, non‐spore‐forming bacteria commonly found in various environments, including soil (18% of the NCBI‐Biosample data) and aquatic environments (36% of the NCBI‐Biosample data), all over the world. Additionally, the *Paracoccus* genus has been previously identified as one of the most abundant genera present in cultural heritage artefacts, including cave and grotto wall paintings, as well as historical books and textiles (Ma et al. 2015; Pietrzak et al. 2017; Saridaki et al. 2022; Pavlović et al. 2023). However, this genus remains relatively understudied; indeed, according to the NCBI‐Biosample database, only three taxa associated with mural paintings have been identified at the species level: *P. cavernae* , isolated from Ardales Cave, Spain (Dominguez‐Moñino et al. 2016); *Paracoccus onubensis*, obtained in Gruta de las Maravillas, in Huelva, Spain (Gutierrez‐Patricio et al. 2021); *Paracoccus tibetensis* recorded in a 1500‐year‐old tomb in Shanxi Province, China (Zhu et al. 2013). The presence of *Paracoccus* species on mural walls is not surprising due to their metabolic versatility, including the ability to oxidize sulphur compounds and to produce biofilm to degrade various organic compounds and survive in nutrient‐poor environments (Puri et al. 2022).

In this study, the occurrence of *P. marcusii* IBR3 suggests that even lesser‐known bacterial taxa may play an important role in the alteration of the mural painting surfaces. In fact, the biochemical activities of *P. marcusii* IBR3 not only facilitate microbial colonization but also may actively drive the physical and chemical deterioration of mural paintings. Hydrolysis of casein and gelatin leads to the formation of acidic byproducts, which can destabilize the paint layer by weakening binding agents and thus damaging pigments and underlying plaster. Similarly, the dissolution of calcium carbonate decreases the local pH, promoting acidification that can damage pigments and the mural's substrate and can lead to the formation of calcium carbonate precipitates, affecting visual and structural integrity. The contribution of *P. marcusii* to the deterioration of mural artworks, either directly through enzymatic degradation of organic binders or indirectly by altering the local microenvironment, may have been underestimated and requires the development of targeted cleaning and control strategies.

Several studies have investigated the antimicrobial properties of essential oils against diverse microorganisms implicated in the biodeterioration of cultural heritage materials preserved in archives, libraries, and museums (Borrego et al. 2010, 2014; Bosco et al. 2022; Russo and Palla 2023; Fernandes et al. 2025). Lavin et al. (2016) showed that the vapours of the essential oils of *Thymus vulgaris* reduced or stopped the growth of *Scopulariopsis* spp. and *Fusarium* spp. isolated from historical archives, to cite only a few examples. This study reports for the first time the efficacy of the *T. serpyllum* essential oil against *P. marcusii*, based on the results of a preliminary in vitro evaluation. Because these findings derive exclusively from in vitro assays, they should be interpreted as a proof of concept rather than as direct evidence for conservation application. The present study does not assess possible interactions with pigments, binders, or plaster substrates, nor does it address treatment persistence, surface compatibility, or application procedures under real conditions. Therefore, any conservation implications remain preliminary and should be validated in mock‐up systems and ultimately, in situ studies. Within these limits, the MIC, MBC, and ED50 values (0.4, 1.0, 0.37 mg/mL), together with cell membrane integrity and permeability assays and ATR‐FTIR analysis, indicated that Thy‐EO exhibited effective biological activity at relatively low concentrations. Although the increase in crystal violet uptake was biologically modest, this assay should be interpreted as a complementary indicator of altered membrane permeability rather than as a stand‐alone measure of antibacterial efficacy. In contrast, the dose‐dependent increase in nucleic acid and protein leakage, together with the clear separation between treated and untreated samples in the ATR‐FTIR PCA, supports the biological relevance of the membrane damage induced by Thy‐EO. The significant alterations detected in OH and CH stretching regions, amide I–II bands, and polysaccharide‐associated peaks further support a mechanism involving destabilization of membrane lipids and proteins. These observations align with previous studies reporting the capacity of essential oils to integrate into lipid membranes due to their hydrophobic nature, thereby increasing permeability and compromising membrane integrity (Yap et al. 2021; Al‐Mijalli et al. 2023). Consistently, Xu et al. (2008) demonstrated that carvacrol and thymol at 200 mg/mL markedly increased membrane permeability, while Kauser et al. (2024) showed that thymol at 32–125 μg/mL inhibited the growth of several *Candida* species and altered their morphology.

Overall, our results support the antimicrobial potential of *T. serpyllum* EO and reinforce previous reports documenting its efficacy against a wide range of bacterial and fungal taxa (Huleihel et al. 2009; Devi et al. 2010; Vettraino et al. 2023; Di Rosario et al. 2024).

*Thymus serpyllum* EO used in this study was characterized by a high proportion of carvacrol (51.88%) and p‐cymene (40.28%). Several studies report that *T. serpyllum* exhibits multiple chemotypes, most commonly thymol‐, carvacrol‐, or p‐cymene‐rich, whose composition varies according to geographic origin, harvest period, genetic factors, and environmental conditions (Casiglia et al. 2019; El Yaagoubi et al. 2021; Özay and Pehlivan 2024). This compositional variability may influence antimicrobial performance and should therefore be considered when evaluating reproducibility and comparing results across studies. For this reason, we have included full GC–MS characterization of the batch used in this work, and future applications would benefit from the use of chemically profiled EO batches to ensure more consistent biocidal performance.

## 5 Conclusions

This study reports the isolation and identification of *P. marcusii* IBR3 from a deteriorated 19th‐century mural painting in a private Italian villa, marking a significant advancement in the field of cultural heritage microbiology. Although species belonging to the *Paracoccus* genus have been sporadically associated with cultural heritage artefacts, the species *P. marcusii* had not previously been identified in this context. Furthermore, this study demonstrated that essential oil extracted from *T. serpyllum* had potent inhibitory activity against *P. marcusii* IBR3 in in vitro tests, by inducing extensive membrane destruction, leading to inhibited bacterial growth. These results highlight the use of *T. serpyllum* –derived oil as an eco‐friendly alternative to conventional chemical treatments for preventing biodeterioration of mural paintings. However, since the present work was conducted exclusively in vitro, these findings should be considered preliminary until validated on representative material mock‐ups and under real conservation conditions.

## Author Contributions

**Anna Maria Vettraino:** conceptualization, investigation, methodology, data curation, supervision, resources, writing – review and editing, writing – original draft. **Vittorio Vinciguerra:** investigation, methodology, writing – review and editing. **Michele Narduzzi:** investigation, writing – review and editing. **Claudia Pelosi:** investigation, writing – review and editing, methodology. **Chiara Antonelli:** investigation, writing – review and editing.

## Conflicts of Interest

The authors declare no conflicts of interest.

## Data Availability

The data that support the findings of this study are available from the corresponding author upon reasonable request.
